# Primary malignant pericardial mesothelioma: a case report

**DOI:** 10.11604/pamj.2020.36.375.25336

**Published:** 2020-08-31

**Authors:** Sameh Ben Farhat, Maroua Salah, Sami Milouchi

**Affiliations:** 1 Cardiology Department, Habib Bourguiba University Hospital, Medenine, Tunisia

**Keywords:** Pericardial effusion, malignancy, pericardial mesothelioma

## Abstract

Metastases to the heart and pericardium are much more common than primary malignant neoplasms. Primary malignant pericardial mesothelioma is a rare tumor that arises from the mesothelial cells of the pericardium. It is usually characterized by a delayed diagnosis, a low response to treatment, and a poor prognosis with an overall survival up to six months after the onset of symptoms. We report a rare case of a 32-year-old woman with primary pericardial malignant mesothelioma that was diagnosed 4 months after the onset of pericardial effusion as the first clinical manifestation.

## Introduction

Primary malignant pericardial mesothelioma (PPM) is an exceedingly rare neoplasm that accounts for about 4% of primary heart and pericardial tumors [1]. There are at present approximately 200 cases reported in the literature [2]. Because of its non-specific and insidious clinical presentation, a definitive diagnosis can be challenging and is often delayed [2]. Common clinical symptoms typically include heart failure, pericardial effusion, and angina pectoris [2]. Non-invasive imaging modalities are useful to aid earlier diagnosis and guide biopsies [3]. However, histopathological examination remains the gold standard for definitive diagnosis [4]. No consensus on treatment strategy has been established yet, and these tumors are still associated with low survival rates and a grim prognosis [4].

## Patient and observation

**Figure 1:**
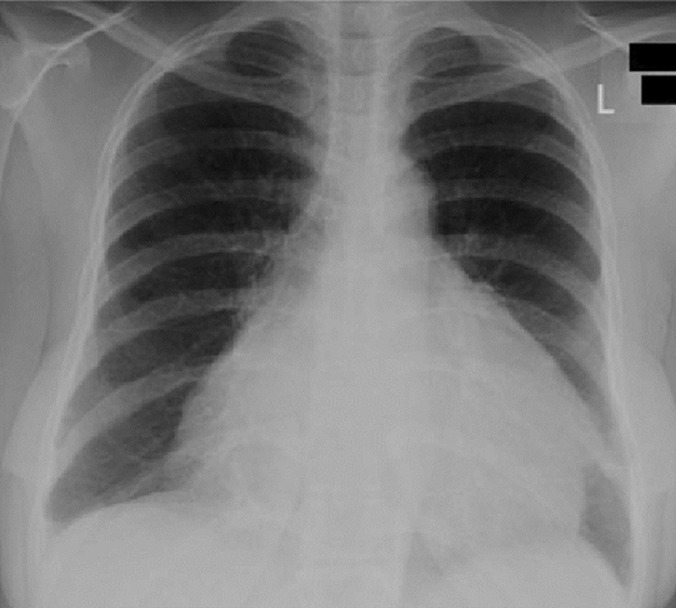
chest radiography demonstrating an enlarged heart shadow

A 32-years-old woman without significant past medical history was hospitalized in our department with 15 days history of shortness of breath. Physical examination was unremarkable, and the patient denied any exposure to asbestos. Electrocardiogram (ECG) showed sinus rhythm. There were no conduction or repolarization abnormalities. Chest X-ray showed an enlarged heart (Figure 1). Transthoracic echocardiography (TEE) revealed a mild pericardial effusion without signs of cardiac tamponade and with preserved biventricular function. The diagnosis of acute pericarditis was retained, and the patient was discharged in a stable condition with colchicine and ibuprofen.

**Figure 2:**
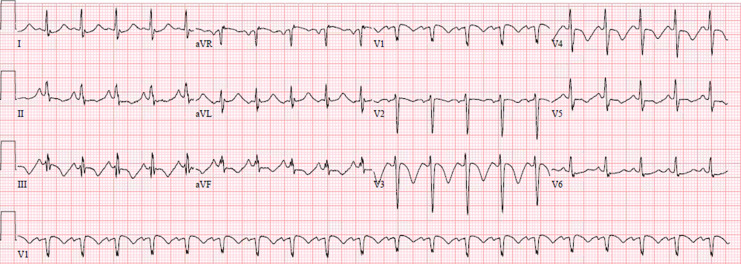
standard 12 leads ECG showing sinus rhythm tachycardia, signs of right atrial dilation and negative T waves in inferior and antero-septo-apical leads

**Figure 3:**
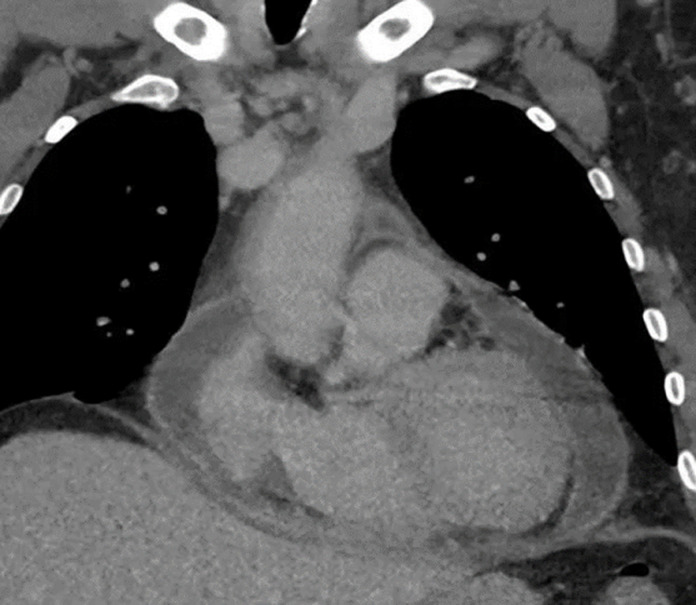
computed tomography scan of the chest showing the fluid collection within the pericardial sleeves

A month later, she was readmitted for chest pain. On physical examination, she has not been febrile and there were no heart murmur or signs of pulmonary congestion. Her central venous pressure was normal and there was no pedal edema. Vital signs showed a blood pressure of 96/55 mmHg, a pulse of 110 beats per minute, and peripheral oxygen saturation of 98%. ECG revealed sinus rhythm tachycardia with negative T waves in inferior and antero-septo-apical leads (Figure 2). TEE showed a worsening of the pericardial effusion but with no signs of right ventricular chambers collapse. We further noted an incipient systolic right ventricular dysfunction. Creatinin, troponin and NT pro-BNP levels were within normal ranges. Hepatic and thyroid functions were normal. Inflammatory markers were elevated with a c reactive protein level of 166 mg/L. Blood count revealed anemia. Cardiac tomography (CT) scan showed a circumferential pericardial effusion with no pericardial thickening or other abnormalities (Figure 3).

**Figure 4:**
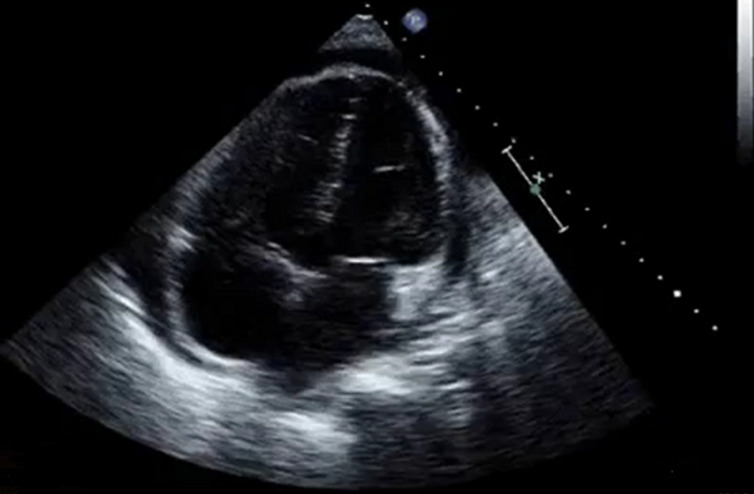
echocardiogram demonstrating circumferential pericardial effusion with thickening of the myocardium of the right ventricular wall

Auto-immune pericarditis was unlikely as we did not detect positive rheumatoid factor, cryoglobilins, antinuclear, and anti-neutrophil cytoplasmic antibodies. Further investigations including serology for viruses commonly incriminated in pericarditis, human immunodeficiency virus, brucellosis, salmonellosis, toxoplasmosis, fever Q and Lyme disease as well as tuberculin skin test were also negative. Thus, a definitive diagnosis could not be obtained, so we opted for pericardiocentesis. Cytological examination showed subacute inflammation with lympho-plasmacytic predominance and mesothelial hyperplasia, but without any signs of malignancy. The patient was treated for suspected tuberculous pericarditis. Six weeks later, with the decrease in corticosteroid therapy, she consulted for recurrence of the same symptomatology. TEE was performed and showed a pericardial effusion with paradoxical septal motion (Figure 4). She was scheduled then for a thoracoscopic surgical biopsy. Histopathological examination of the specimen confirmed the diagnosis of malignant mesothelioma with right ventricular myocardial invasion. On immuno-histo-chemistry, neoplastic cells were strongly positive for calcretinin and WT1 protein. We referred her for palliative chemo and radiotherapy, but she died of refractory heart failure 4 months following the definitive diagnosis.

## Discussion

Primary pericardial mesothelioma accounts for about 4% of the primary heart and pericardial tumors [1]. There is a slight male predominance with an average age at presentation of 46 years [2]. Unlike pleural mesothelioma, it has not been found to be associated with exposure to asbestos [4]. The onset of symptoms is usually insidious. Clinical features are non-specific but commonly include pericardial syndromes, heart failure, and angina pectoris [2]. Echocardiographic findings are also non-specific. They may show pericardial effusion, tamponade, thickening of the pericardium, or even a pericardial mass [3]. It further allows functional cardiac evaluation and the assessment of wall motion abnormalities [3]. Magnetic resonance imaging and CT may prove useful in showing the degree of constriction and the extent of the tumor to the adjacent structures [2, 5]. The definitive diagnosis can be challenging and most cases (≈ 75%) are diagnosed post mortem [6]. PPM are often misdiagnosed as other causes of acute pericarditis, but the most important differential diagnosis remains tuberculous pericarditis [2]. The presence of relapsing cardiac tamponade or persistent worsening paricarditis and the resistance to effective anti-inflammatory or anti-tuberculous therapy should draw the attention of physicians and incite them to perform a pericardial tissue biopsy [2].

Diagnostic pericardiocentesis is often non-conclusive. In a literature review of Nilsson *et al*. only 23% of cytological analysis were found to be malignant [7]. Thus, histopathological examination and immunohistochemistry of specimens drawn from pericardiectomy, pericardial tumor biopsy, or autopsy are mandatory to establish the definitive diagnosis [8]. To date, there is no consensus on treatment [4]. Surgery remains palliative in most cases and complete tumor excision is done scarcely [1]. Response to radiotherapy is generally poor [4]. Chemotherapy agents reduce tumor mass and progression and may even prolong survival [4]. Despite optimized therapy, survival after the definitive diagnosis does not exceed 6 months [2].

## Conclusion

This case report highlights the difficulties encountered by physicians in the diagnosis of primary malignant pericardial mesothelioma. Neoplastic causes should be considered in the differential diagnosis of acute pericarditis, especially in case of resistance to conventional therapy.
